# Repeatability and reproducibility of cerebral ^23^Na imaging in healthy subjects

**DOI:** 10.1186/s12880-019-0324-6

**Published:** 2019-04-03

**Authors:** Melissa M. Meyer, Stefan Haneder, Simon Konstandin, Johannes Budjan, John N. Morelli, Lothar R. Schad, Hans U. Kerl, Stefan O. Schoenberg, Christoph Kabbasch

**Affiliations:** 1 Institute of Clinical Radiology and Nuclear Medicine, Medical Faculty Mannheim, University of Heidelberg, Theodor-Kutzer-Ufer 1-3, 68167 Mannheim, Germany; 2 Institute of Diagnostic and Interventional Radiology, University Hospital Cologne, University of Cologne, Cologne, Germany; 3 MR Physics, Fraunhofer MEVIS, Bremen, Germany; 4 St. John’s Medical Center, 1923 South Utica Ave, Tulsa, OK 74104 USA; 5 Department of Computer Assisted Clinical Medicine, Heidelberg University, Mannheim, Germany; 6 Department of Neuroradiology, University Medical Center Mannheim, Heidelberg University, Mannheim, Germany

**Keywords:** ^23^Na (sodium) imaging, Oncologic imaging, Cerebral magnetic resonance imaging

## Abstract

**Background:**

Initial reports of ^23^Na magnetic resonance imaging (MRI) date back to the 1970s. However, methodological challenges of the technique hampered its widespread adoption for many years. Recent technical developments have overcome some of these limitations and have led to more optimal conditions for ^23^Na-MR imaging. In order to serve as a reliable tool for the assessment of clinical stroke or brain tumor patients, we investigated the repeatability and reproducibility of cerebral sodium (^23^Na) imaging in healthy subjects.

**Methods:**

In this prospective, IRB approved study 12 consecutive healthy volunteers (8 female, age 31 ± 8.3) underwent three cerebral ^23^Na-MRI examinations at 3.0 T (TimTrio, Siemens Healthineers) distributed between two separate visits with an 8 day interval. For each scan a T1w MP-RAGE sequence for anatomical referencing and a 3D-density-adapted, radial GRE-sequence for ^23^Na-imaging were acquired using a dual-tuned (^23^Na/^1^H) head-coil. On 1 day, these scans were repeated consecutively; on the other day, the scans were performed once. ^23^Na-sequences were reconstructed according to the MP-RAGE sequence, allowing direct cross-referencing of ROIs. Circular ROIs were placed in predetermined anatomic regions: gray and white matter (GM, WM), head of the caudate nucleus (HCN), pons, and cerebellum. External ^23^Na-reference phantoms were used to calculate the tissue sodium content.

**Results:**

Excellent correlation was found between repeated measurements on the same day (r^2^ = 0.94), as well as on a different day (r^2^ = 0.86). No significant differences were found based on laterality other than in the HCN (63.1 vs. 58.7 mmol/kg WW on the right (*p* = 0.01)). Pronounced inter-individual differences were identified in all anatomic regions. Moderate to good correlation (0.310 to 0.701) was found between the readers.

**Conclusion:**

Our study has shown that intra-individual ^23^Na-concentrations in healthy subjects do not significantly differ after repeated scans on the same day and a pre-set time interval. This confirms the repeatability and reproducibility of cerebral ^23^Na-imaging. However, with manual ROI placement in predetermined anatomic landmarks, fluctuations in ^23^Na-concentrations can be observed.

## Background

Initial reports of ^23^Na magnetic resonance imaging (MRI) date back to the 1970s [1]. However, methodological challenges of the technique – low SNR due to low in-vivo sodium concentrations and low available field strengths – hampered its widespread adoption for many years. Recent technical developments such as clinical 3 Tesla MRI, improved coils, and faster gradients have overcome some of these limitations and have led to more optimal conditions for ^23^Na-MR imaging. Several recent publications have demonstrated the feasibility of ^23^Na-MRI for in-vivo imaging of physiological conditions in the kidney [2–4], cartilage and musculoskeletal system [5–8], heart [9–11], and prostate [12].

The use of ^23^Na-MRI for imaging of the brain was first described in 1983 by Hilal et al., who observed changes following an induced stroke in a cat model [13]. In 1987, Turski et al. utilized the technique in patients with a cerebral neoplasm [14]. Since then, several feasibility studies [15, 16] have utilized ^23^Na-MRI for the imaging of stroke [17–19], demyelinating diseases [20], and brain tumors. In the latter case, various brain tumors exhibit increased sodium concentrations relative to normal brain structures [21–23]. Theoretically, this observation is based on the presence of cell membrane depolarization prior to cell division. Since cell division is increased in neoplasia, intracellular sodium concentration (ISC) is thus increased and consequently the total ^23^Na-concentration rises [24]. Therefore, ^23^Na-MRI could be useful for predicting therapeutic tumor response in brain [22] or other organs [25].

In order to serve as a reliable tool for the assessment of clinical stroke or brain tumor patients, studies have to investigate the absolute levels and differences of sodium concentrations in healthy volunteers undergoing consecutive MRIs with a dual-tuned (^23^Na/^1^H) head-coil at different time points. Thus, the objective of this study was to verify the repeatability and reproducibility of cerebral ^23^Na-MR imaging in healthy subjects.

## Methods

This study was performed without any financial or non-financial support from industry. The authors had complete control of the data submitted for publication. The local institutional review board (IRB) and ethics committee (Medical Ethics Committee II Mannheim, Germany; reference number: 2013 566 N – MA) approved this prospective baseline study which was performed in accordance with the ethical standards laid down in the 1964 Declaration of Helsinki and its later amendments.

### Healthy volunteers

After the procedure was fully explained, written informed consent was obtained from all participants prior to MR imaging. Twelve healthy volunteers (8 females, 4 males, mean age 31 ± 8.3 years) underwent three brain MRI examinations (V1–3,) spread between two separate visits with an interval of 8 days between. During one visit, two consecutive, repetitive scans (V1 and V2) were obtained. During a time gap of 5 min in between the two scans the subject was moved out of the gantry. The isocenter was identical to the prior examination using the scanner’s dedicated laser positioning device. At the second visit, only one examination was performed. The subject was positioned in a similar position according to anatomic landmarks and the isocenter was again set using the scanner’s dedicated laser positioning device. The group was subdivided randomly, whereby one half of the subjects received two MRI scans during the first visit and one MRI during the second visit. The other half of the subjects received one MRI on the first visit and two MRIs on the second visit. Besides the standard exclusion criteria for MRI (i.e. pacemaker), patients with a history of brain lesions or neurological symptoms were excluded. For both visits, the subjects were scheduled at the same time of the day (to exclude influences of circadian rhythm changes of sodium concentrations [26]) and were asked to abstain from the consumption of alcohol and coffee for eight hours prior to the exams.

### Brain MRI

All MR examinations were performed on a 3.0 T MR-system (Siemens TimTrio; Siemens Healthineers, Erlangen, Germany). Individuals were positioned supine and head first in a dual-tuned (^23^Na/^1^H) radiofrequency (RF) coil (Rapid Biomedical, Germany). In addition to the survey sequences, the imaging protocol included the following sequence protocol: standard morphological, axial T2 weighted images; axial fluid attenuated inversion recovery (FLAIR) images; diffusion-weighted imaging (DWI) based on echo planar imaging; T1 weighted magnetization-prepared rapid acquisition gradient-echo (MP-RAGE) for anatomical referencing, and a three dimensional (3D)-density-adapted projection reconstruction ^23^Na-sequence [27]. No contrast media was administered. Sequence details are summarized in Table 1. Echo time was defined as the time period between the middle of the excitation pulse and the beginning of the readout. A cylindrical falcon tube with a 0.6% NaCl reference was placed within the field of view as a concentration reference.

**Table 1:** Sequence parameters

	T1w MP-RAGE	3D-DPR-GRE ^23^Na
TR (msec)	1630	120
TE (msec)	3.4	0.2
Projections	NA	17,000
Samples/projection	NA	369
Radial fraction *p*	NA	0.20
Bandwidth	179	NA
Readout time (msec)	NA	20
Inversion time (msec)	900	NA
Flip angle (°)	9	87
Spatial resolution (mm^3^)	1 × 1 × 1	3.6 × 3.6 × 3.6
Field of view (mm^2^)	256 × 256	NA
Sections	160	NA
b-value	NA	NA

*Note: NA* not applicable, *TR* repetition time, *TE* echo time, *MP-RAGE =* magnetization-prepared rapid acquisition gradient-echo*,* 3D-DPR-GRE 3D-density adapted radial gradient echo

### Quantification of sodium concentration

**Fig. 1:**
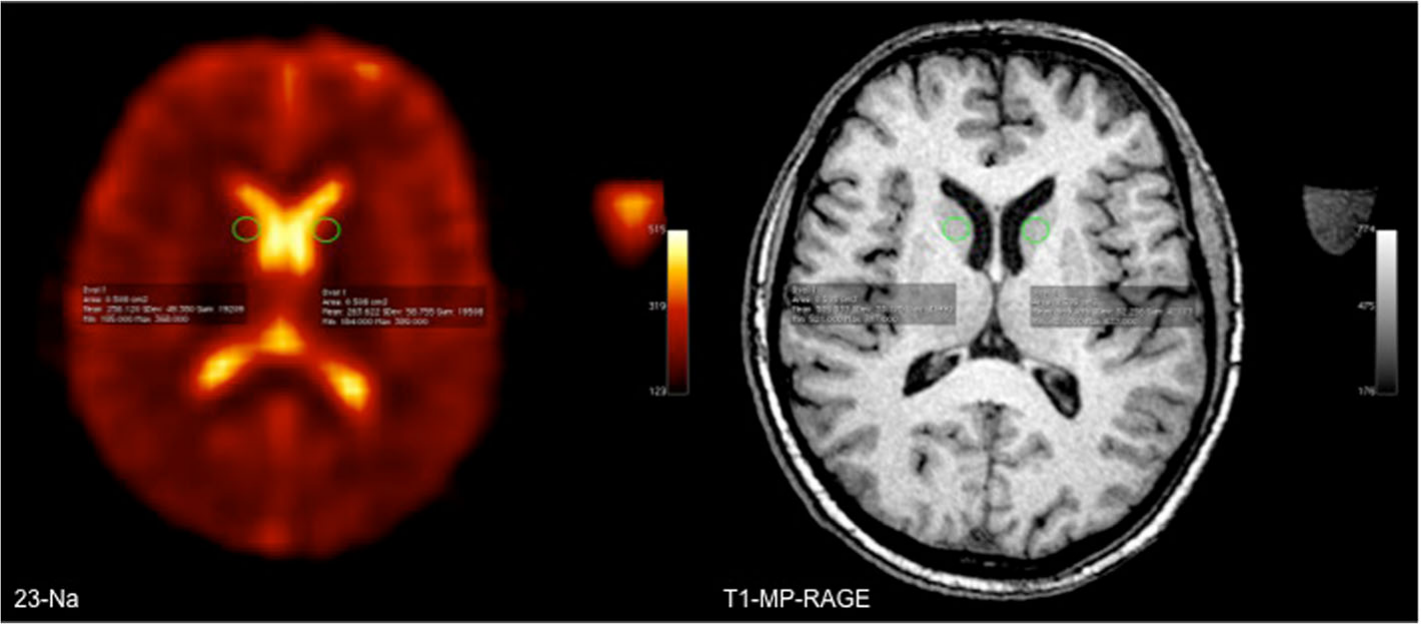
*Left*: Example of region-of-interest (ROI) placement in the head of the caudate nucleus on a ^23^Na-image; *right*: T1 MP-RAGE as an anatomical reference image

^23^Na-MRI images were co-registered offline to the corresponding T1 weighted MP-RAGE image for each volunteer using the statistical parametric mapping toolbox (SPM8, The Wellcome Trust Centre for Neuroimaging, London) of MATLAB (R2010a, The MathWorks Inc., Natick, MA). Data sets of every patient were analyzed on an OsiriX DICOM viewer 3.8.1 (OsiriX Foundation, Geneva, Switzerland). A quantitative assessment of ^23^Na-concentration was performed by two radiologists (reader 1 and 2 with 6 and 2 years experience in ^23^Na-imaging, respectively) who independently, manually placed predetermined, identically-sized (0.508 cm^2^) circular region of interests (ROIs) on three consecutive slices according to the following specific anatomic landmarks: the bilateral central gray (centrum semiovale) and temporal white matter (GM/WM), the bilateral heads of the caudate nuclei, the pons at the level of the superior cerebellar pedunculi, and the bilateral cerebellum within the pedunculi cerebelli medius. These ROIs were placed on every examination. An example of a ROI placement is shown in Fig. 1. The ROIs were placed on the morphological ^1^H images (MP-RAGE) and afterwards copied onto the ^23^Na-images. Values for ^23^Na-concentration in millimoles per kilogram wet weight were calculated according to the following formula:

$$ {\left[ Na\right]}_{tiss}=\frac{S^{Na\  tiss}}{S^{Na\  ref}}{\left[ Na\right]}^{Ref}x\ \frac{1-\exp\ \left(-\frac{TR}{T_1^{ref}}\right)}{1-\exp \left(-\frac{TR}{T_1^{tiss}}\right)}\ x\ \frac{alfa\ x\exp \left(-\frac{TE}{T_{2f}^{ref}}\right)+ beta\ x\ \exp \left(-\frac{TE}{T_{2s}^{ref}}\right)}{alfa\ x\exp \left(-\frac{TE}{T_{2f}^{tiss}}\right)+ beta\ x\ \exp \left(-\frac{TE}{T_{2s}^{tiss}}\right)} $$

where [Na] is sodium concentration, tiss is tissue, ref. is reference phantom, S^Na^ is signal intensity of sodium, f is fast, s is slow, TE is echo time, TR is repetition time, alfa/beta is 0.6/0.4 for brain tissue [28] and 0.0/1.0 for CSF.

With:

T2ref/f = 7.15 msec, T2ref/s = 33.7 msec

Brain: T2tiss/f = 4 msec, T2tiss/s = 29 msec and T1tiss/s = 29 msec

CSF: T2tiss/s = 55 msec and T1tiss = 58.1 msec

### Statistical analysis

JMP 10.0 (SAS Institute Inc., Cary, North Carolina, USA) was used for statistical analysis. Descriptive statistics (median, mean, 95% confidence interval and standard deviation (SD)) were used for all data. For the analysis of repeatability and reproducibility of cerebral ^23^Na-concentrations, repeatability was defined as repeated exams on the same day (V1 and V2) with the same experimental setup, whereas reproducibility was defined as a repeated exam (V3) with a pre-set time interval of 8 days. Repeatability and reproducibility analyses were performed using a Pearson’s momentum correlation. Intra-individual ^23^Na-concentrations in specific ROIs were tested with repeated measures ANOVA using a Dunnett’s test. Chi-squared tests were performed for subgroup analysis in terms of the left versus the right hemisphere. *P* values less than 0.05 were considered statistically significant. A normal distribution was confirmed using a Kolmogorov-Smirnov test (0.019 < *p* < 0.622). Due to the data scale, Spearman’s rho tests were chosen to determine the inter-reader correlation.

## Results

### Quantification of ^23^Na-concentration

**Fig. 2:**
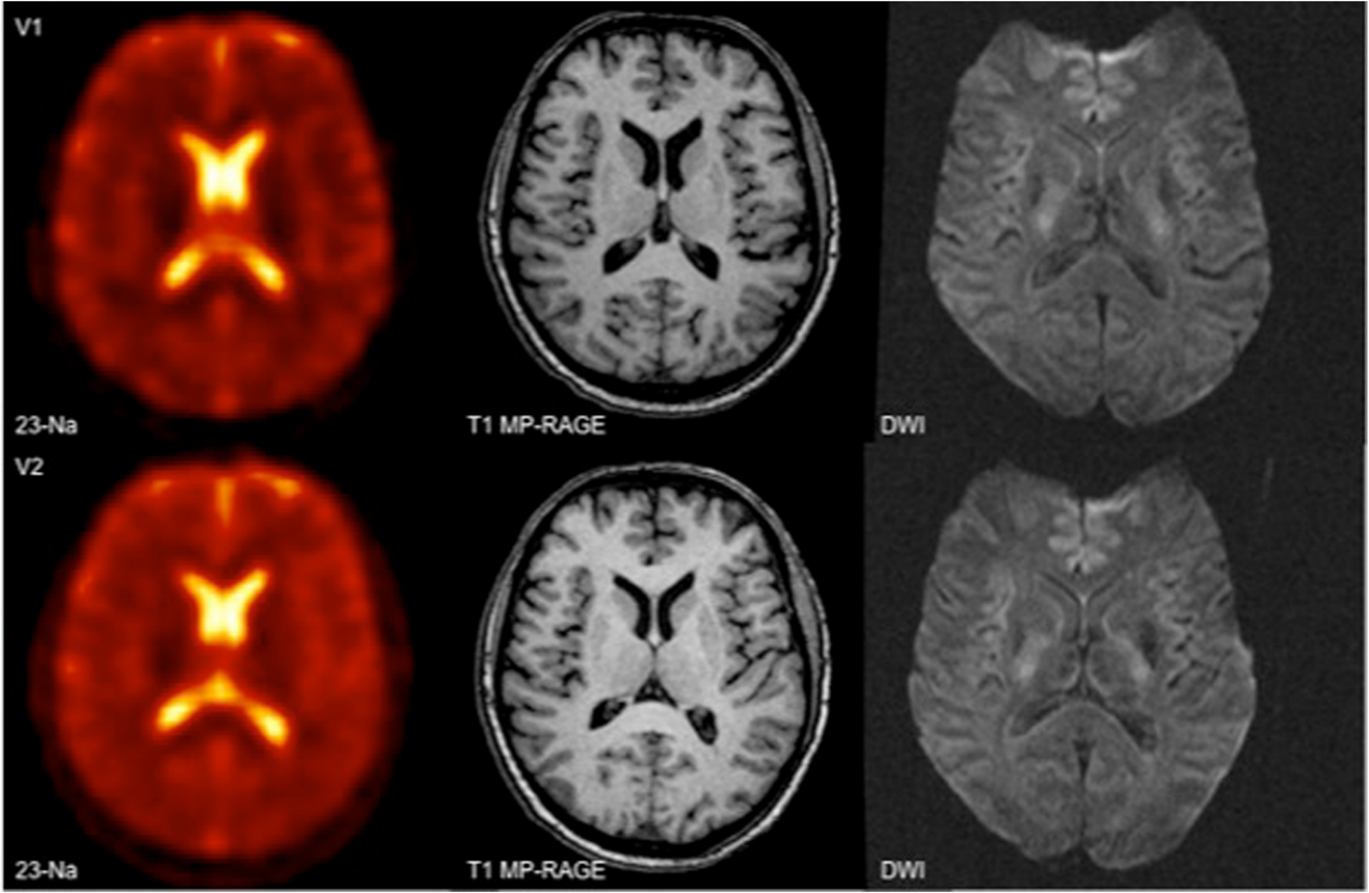
*Top*: First examination (V1) on the first examination day with ^23^Na-image, T1 MP-RAGE and DWI (from left to right); *bottom*: Second examination (V2) on the first examination day

The overall mean ^23^Na-concentration (in millimoles per kilogram wet weight) for both readers and sides was 51.5 ± 4.5 mmol/kg WW and 40.9 ± 3.8 mmol/kg WW for the respective gray and white matter, 60.9 ± 8.1 mmol/kg WW for the head of the caudate nucleus, 39.8 ± 5.3 mmol/kg WW for the pons, and 40.1 ± 4.9 mmol/kg WW for the cerebellum. Subgroup analysis comparing the left and right hemisphere did not reveal statistically significant differences other than in the head of the caudate nucleus. ^23^Na-concentrations of the head of the caudate nucleus were 63.1 ± 8.4 mmol/kg WW on the left compared to 58.7 ± 7.3 mmol/kg WW on the right (*p* = 0.01) (Fig. 2).

### Inter-individual ^23^Na-fluctuations

Pronounced inter-individual differences were identified in all anatomic regions. The max/min ^23^Na concentration values and maximum percentage differences were: Gray matter = 65.4/42.4 mmol/kg WW (35.1%), white matter 52.9/34.5 mmol/kg WW (34.7%), caudate head = 81.5/49.5 mmol/kg WW (39.3%), pons = 58.4/33.2 mmol/kg WW (43.0%), and cerebellum 52.3/33.0 mmol/kg WW (36.9%).

### Repeatability analysis: intra-individual ^23^Na-concentrations

**Fig. 3:**
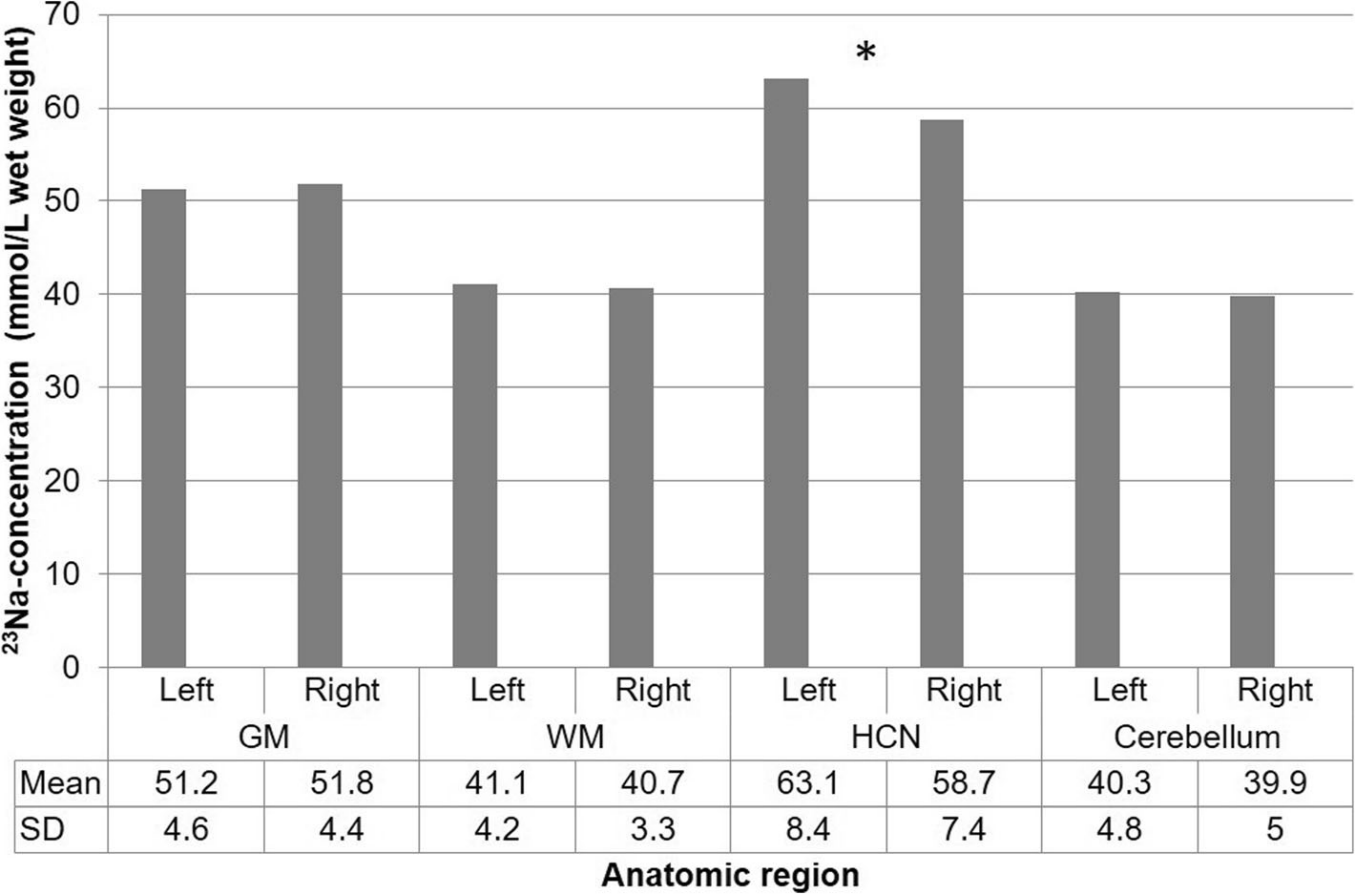
Total ^23^Na-concentrations with standard deviations (SD) according to laterality (GM = gray matter; WM = white matter; HCN = head of the caudate nucleus); * indicates significant difference with *p* = 0.01

Excellent correlation (r^2^ = 0.94) was found between repeated measurements on the same day (V1 and V2). An example of two consecutive images is shown in Fig. 3. Overall, the mean ^23^Na-concentration measured at V1 and V2 were not significantly different (V1: 52.7 ± 20.7 mmol/kg WW vs V2: 53.2 ± 20.8 mmol/kg WW; *p* > 0.05). Similarly, there was no significant difference across all anatomic regions between measurements at V1 and V2 (compare Table 2; *p* > 0.05). The order of the MRI examinations (two examinations at the first or the second appointment) had no significant influence on the ^23^Na-concentration (0.206 < *p* < 0.886).

**Table 2:** Repeatability and Reproducibility of ^23^Na concentrations

*TSC*	*Repeatability*			*Reproducibility*		
Region	V1	V2	*p*-value	V1	V3	*p*-value
GM	51.0 ± 6.3	51.5 ± 6.7	0.77	51.0 ± 6.3	52.0 ± 7.2	0.72
WM	40.5 ± 4.8	41.2 ± 4.4	0.30	40.5 ± 4.8	41.0 ± 6.3	0.83
HCN	59.9 ± 9.9	61.1 ± 9.7	0.54	59.9 ± 9.9	61.7 ± 10.9	0.41
Cerebellum	40.5 ± 7.7	40.6 ± 5.0	0.44	40.5 ± 7.7	39.2 ± 5.7	0.39
Brainstem	41.1 ± 10.2	39.4 ± 6.8	0.80	41.1 ± 10.2	40.0 ± 7.9	0.93
CSF	102.1 ± 21.6	103.8 ± 20.9	0.93	102.1 ± 21.6	103.6 ± 24.5	0.98
Mean	52.7 ± 20.7	53.2 ± 20.8	0.68	52.7 ± 20.7	53.1 ± 21.6	1.0

Note: TSC = total sodium concentration; V1/V2 = repeated exams on the same day; V1/V3 = exam on different dates

### Reproducibility analysis: intra-individual ^23^Na-concentrations

Excellent correlation (r^2^ = 0.86) was found between V1 and V3 measurements. Overall, mean ^23^Na-concentrations measured at V1 and V3 were not significantly different (V1: 52.7 ± 20.7 mmol/kg WW vs V2: 53.1 ± 21.6 mmol/kg WW; *p* > 0.05). Similarly, there were no significant differences across the anatomic regions between measurements of V1 and V3 (compare Table 2; *p* > 0.05). When comparing the maximum and the minimum values for the ^23^Na-concentration, the mean percentage of intra-individual differences ranged from 6.5% (GM) and 10% (Pons).

### Inter-reader agreement

**Fig. 4:**
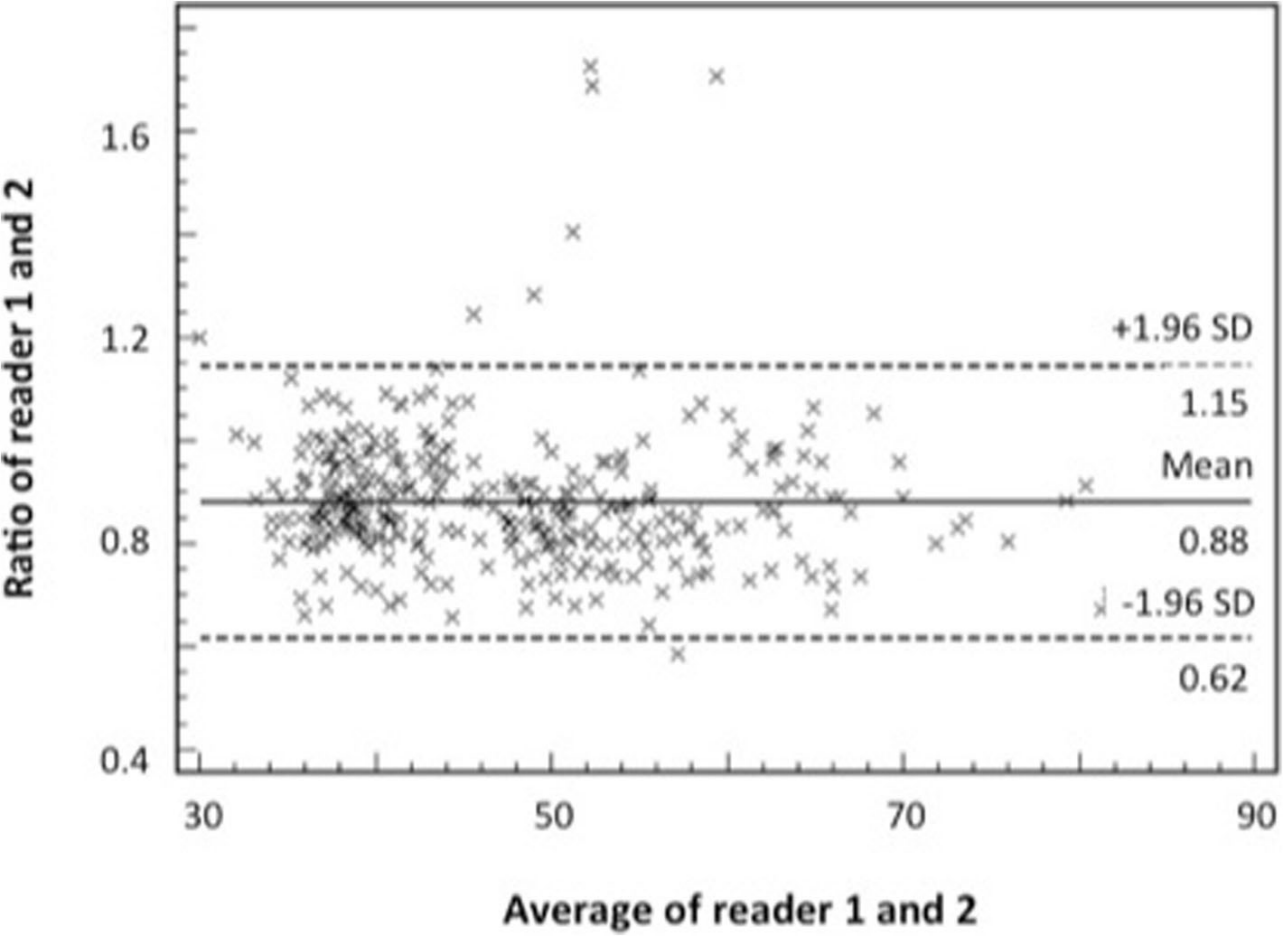
Bland-Altman plot, plotting the ratio of the two readers versus the average of both readers. The lines and given numbers on the right side represent the mean of the ratio and the +/− 1.96 standard deviation (SD) of the ratio

Moderate to good correlation (0.310 to 0.701) was found between the two readers. Overall, the mean ^23^Na-concentration as measured for the different anatomic regions by reader 2 was greater than that measured by reader 1 (see Fig. 4). The largest discrepancies were seen in the gray matter and in the head of the caudate (47.4 vs. 55.5 mmol/kg WW and 56.0 vs. 65.8 mmol/kg WW, respectively; *p* > 0.05).

## Discussion

The potential of ^23^Na-MRI for brain imaging in oncological and non-oncological applications remains to be ultimately determined [29]. To further implement ^23^Na-MRI in clinical neuroimaging, particularly in the setting of oncological follow-up, certain fundamental requirements are to be fulfilled. One of these is the intra-individual reliability and reproducibility of brain ^23^Na-concentrations on consecutive MR examinations. Little published data are available regarding the ^23^Na-concentrations of healthy gray and white matter, but the values of the current study are in the range described in the literature [20, 21, 30]. This study showed no significant asymmetries in the ^23^Na-concentrations of several predefined areas of the human brain, other than between the left and right caudate head. These differences could be attributed to partial volume effects of the immediately adjacent cerebrospinal fluid (CSF). The relatively narrow head of the caudate nucleus impeded the manual ROI placement, which could explain these differences. One way to potentially solve this problem is by normalization of the data to a reference brain data set, as done in psychiatry studies for hippocampus volume measurements and the use of semi-automatic segmentation tools. Statistically significant intra-individual differences were neither seen between two consecutive MR exams nor between MR exams separated temporally by 8 days. This supports the repeatability and reproducibility of cerebral ^23^Na-MRI. However, per-patient differences in sodium concentration were as high as 10%, depending on the anatomic region. This potential bias should be considered in any further longitudinal or follow up study. Nevertheless, future studies may benefit from an intra-individual longitudinal approach, since inter-individual differences at a single time point can reach over 30%. Another important consideration for the clinical implementation of brain ^23^Na-MRI would be the development of a simple evaluation method for ^23^Na-MRI imaging with sufficient inter-reader reliability. The inter-reader reliability in this study was moderate but not perfect; however, this may be explained in part by the differences in clinical ^23^Na-MRI experience between the readers (6 vs. 2 years). As previously mentioned, a semi-automatic segmentation tool could facilitate this assessment and decrease inter-reader variability. In general, several future avenues may benefit from a broader clinical implementation of ^23^Na-MRI. Accuracy and quantification of ^23^Na concentrations between different research facilities, hospitals, and techniques (e.g. UTE, radial or cones scheme [31]) should be confirmed. Double-tuned coils seem to be the ideal instrument for ^23^Na-imaging enabling the appropriate fusion of ^1^H- and ^23^Na-images. Although the reliability of the current technique appears to be sufficient, this potential (small) for bias should be taken into account within further studies. While intra-individual comparison seems acceptable for assessment of ^23^Na concentration, biases may arise due to natural inter-individual differences, and further follow-up studies are necessary. Finally, future semi-automatic segmentation tools as proposed could help to partially overcome biases due to inter-reader interpretative differences.

## Limitations

Our study had a number of limitations. Since it was initiated as a baseline study, the study group was relatively small and young without any known medical conditions. To evaluate the true clinical value of this technique, further evaluations in longitudinal studies will be necessary both in healthy volunteers as well as in patients.

## Conclusion

Our study has shown that intra-individual ^23^Na-concentrations in healthy subjects do not significantly differ after repeated scans on the same day and a pre-set time interval. This confirms the repeatability and reproducibility of cerebral ^23^Na-imaging and serves as a baseline for future studies. However, with manual placement of ROIs in predetermined anatomic landmarks, fluctuations in ^23^Na-concentrations can be observed. This must be taken into account if ^23^Na-imaging is to be considered as a potential tool for therapeutic monitoring.

## Abbreviations

DWI: Diffusion-weighted imaging
FLAIR: Fluid attenuated inversion recovery
GM, WM: Gray and white matter
HCN: Head of the caudate nucleus
IRB: Institutional review board
ISC: Intracellular sodium concentration
MP-RAGE: Magnetization-prepared rapid acquisition gradient-echo
MRI: Magnetic resonance imaging
RF: Radiofrequency
SD: Standard deviation
